# Enterovirus D68 and other enterovirus serotypes identified in South African patients with severe acute respiratory illness, 2009–2011

**DOI:** 10.1111/irv.12444

**Published:** 2017-02-26

**Authors:** Orienka Hellferscee, Florette K. Treurnicht, Stefano Tempia, Ebrahim Variava, Halima Dawood, Kathleen Kahn, Adam L. Cohen, Marthi Pretorius, Cheryl Cohen, Shabir A. Madhi, Marietjie Venter

**Affiliations:** ^1^ National Institute for Communicable Diseases of the National Health Laboratory ServiceJohannesburgSouth Africa; ^2^ University of the WitwatersrandJohannesburgSouth Africa; ^3^ Centres for Disease Control and PreventionAtlantaGeorgiaUSA; ^4^ Pietermaritzburg Metropolitan HospitalPietermaritzburgSouth Africa; ^5^ CaprisaUniversity of KwaZulu‐NatalPietermaritzburgSouth Africa; ^6^ University of PretoriaPretoriaSouth Africa

**Keywords:** enterovirus, EV‐D68, pneumonia, South Africa

## Abstract

**Background:**

Human enteroviruses (EV) have been associated with severe acute respiratory illness (SARI) in South Africa.

**Objectives:**

We aimed to describe the molecular epidemiology of EV serotypes among patients hospitalized with SARI during 2009‐2011.

**Patients/Methods:**

Study samples from patients were tested for the presence of enterovirus using a polymerase chain reaction assay.

**Results:**

8.2% (842/10 260) of SARI cases tested positive for enterovirus; 16% (7/45) were species EV‐A, 44% (20/45) EV‐B, 18% (8/45) EV‐C and 22% (10/45) EV‐D. Seventeen different EV serotypes were identified within EV‐A to EV‐D, of which EV‐D68 (22%; 10/45) and Echovirus 3 (11%; 5/45) were the most prevalent.

**Conclusions:**

EV‐D68 should be monitored in South Africa to assess the emergence of highly pathogenic strains.

## 1 Introduction

Pneumonia is a major cause of morbidity and mortality in children worldwide and is responsible for 18% of all deaths in children <5 years of age.^1^ Several respiratory viruses, including enterovirus (EV), have been associated with severe acute respiratory illness (SARI) in South Africa.^2^ Although the majority of EV infections are subclinical, they can lead to a variety of acute and clinical illnesses including upper and lower respiratory tract illness, aseptic meningitis, encephalitis, acute haemorrhagic conjunctivitis, acute flaccid paralysis, myocarditis and neonatal sepsis‐like disease.^3^

EVs are members of the genus *Enterovirus* in the family *Picornaviridae* and have a positive sense single‐stranded RNA genome, 7400‐7500 nucleotides in length.^4^ Human EVs are classified into four species: EV‐A, EV‐B, EV‐C and EV‐D.^5^ This is based on the high sequence diversity within the VP1 capsid region and serotype‐specific neutralization profiles.^6^ Molecular identification methods are crucial for rapid and sensitive EV diagnosis.^6^ EV‐D68 belongs to EV‐D, and until recently, sporadic outbreaks have been reported in Japan, Philippines, the Netherlands and the USA.^7^ Recent reports of severe, fatal disease associated with EV‐D68 infection in the USA renewed interest in EV as cause of severe acute respiratory illness.^8^ EV‐D68 infects primary target tissues of the respiratory tract directly and occasionally infects the central nervous system.^3^ Bayesian phylogenetic analysis of EV‐D68 strains globally revealed the presence of three primary clades (A, B and C) 7, 9, 10 although other literature has separated EV‐D68 into clusters (1, 2 and 3) 11 and lineages (1 and 2).^12^

It is unclear if the recent upsurge in EV‐D68‐associated cases of pneumonia and flaccid paralysis‐like illness are due to true changes in EV‐D68 disease pathogenesis or improved molecular diagnostics.^13^ A previous study from South Africa, using real‐time polymerase chain reaction (PCR) assays, identified EVs in 6% (515/8173) of patients that were hospitalized with pneumonia in South Africa.^14^ However, molecular characterization of these viruses has not been carried out to determine whether some emerging clades might be present in South Africa and the role of specific clades in severe disease in the light of the high HIV seroprevalence in the country. Here we aimed to describe the epidemiology of EV and determine which serotypes were circulating among South African patients hospitalized with SARI during 2009‐2011.

## 2 Materials and Methods

### 2.1 Study design and population

Study samples were obtained from participants enrolled in a prospective hospital‐based surveillance programme for SARI initiated in February 2009, which aimed to describe the aetiology and risk factors for acute lower respiratory tract infection in all age groups in South Africa. The methodology of this study has been described.^14^, ^15^

### 2.2 Sample selection and detection of EV infection

Respiratory specimens (ie nasopharyngeal aspirates for children <5 years of age and nasopharyngeal and oropharyngeal swabs from persons ≥5 years of age) were collected from all enrolled patients, placed in viral transport medium, stored at 4‐8°C and transported to the National Institute for Communicable Diseases (NICD) within 72 hours of collection for testing. Specimens were tested for the presence of 10 respiratory viruses (EV, influenza A and B viruses; parainfluenza virus types 1, 2 and 3; respiratory syncytial virus; adenovirus; rhinovirus; human metapneumovirus) using a multiplex real‐time reverse transcriptase PCR assay which targets the conserved 5` untranslated region of EVs.^14^ Due to challenges with availability of reagents, we did not test for adenovirus from August to October 2011. For this period, retrospective testing was carried out on 30% of randomly selected samples and the prevalence extrapolated. The study period included samples collected from January 2009 to December 2011 (n=10 260). EV‐positive specimens from each year (2009: n=75/315, 2010: n=48/200 and 2011: n=77/327) were randomly selected for genotyping and molecular characterization to obtain 24% of all EV‐positive samples per study year.

### 2.3 Amplification and sequencing of Enteroviruses

The 5′ proximal part of VP1 region was amplified and sequenced, amino acid position 67 to position 133 relative to the start of the VP1 reading frame of the Fermon strain of EV‐D68 (AY426531). Briefly, cDNA synthesis was performed with Transcriptor 1st Strand cDNA Kit (Roche Diagnostics, Mannheim Germany), according to manufacturer's instructions. Primer pairs as described by Nix et al.^16^, 2006 were used. The nested PCR product (375 bp) was analysed on a 1% agarose gel using a 100‐bp molecular weight marker (Roche, Mannheim, Germany) as a size reference. Amplicons were purified using the ExoSAP‐IT enzyme system (USB Corporation, Cleveland OH, USA) and sequenced using the Big Dye terminator version 3.1 cycle Sequencing Ready Reaction kit (Life Technologies, Foster City, CA, USA). Sequences were assembled using Sequencher^®^ version 5 (Gene Codes Corporation , Ann Arbor, MI, USA).

### 2.4 Sequencing analysis

Sequence alignments were performed using MAFFT multiple sequence alignment programme.^17^ Reference sequences included in the final data set were obtained from GenBank. The Kimura‐2 parameter nucleotide substitution model determined as the optimal substitution model using jModelTest was used for the neighbour‐joining (NJ) analysis,^18^, ^19^ and the NJ trees were generated using MEGA 5.2 software.^20^ For this study, we used the A, B and C strain typing nomenclature to describe diversity of EV‐D68 strains.^7^, ^9^, ^10^ Sequences of EV‐D68 partial VP1 genes generated in this study have been deposited in GenBank with the following accession numbers: KX530500‐KX530509. The VP1 nucleotide sequences from EV prototype strains (http://www.picornaviridae.com/enterovirus/enterovirus.htm) used in this study were retrieved from GenBank. The nucleotide sequences for EV‐D68 strains were downloaded from GenBank and represent all known clades.^9^, ^10^

### 2.5 Statistical analysis

Differences in characteristics of EV serotypes were assessed using the Fisher's exact test. *P*‐values <.05 were considered to be statistically significant. Analysis was performed using STATA 13 (Stata Corporation, Texas USA).

### 2.6 Ethical considerations

The SARI protocol was reviewed and approved by the University of the Witwatersrand Human Research Ethics Committee (HREC) and the University of KwaZulu‐Natal Human Biomedical Research Ethics Committee (BREC) protocol numbers M081042 and BF157/08, respectively.

## 3 Results

### 3.1 Clinical and epidemiological characteristics of patients hospitalized with enterovirus‐associated SARI

During the study period, EV was detected in 8.2% (842/10 260) of SARI cases. For observations with complete data, as displayed in Table 1, the majority of EV‐positive patients (86.5%, 726/839) were <5 years of age. EVs were detected throughout the study, although the detection rate was lower in 2010 (5.8%) than in 2009 (8.9%) and 2011 (10.1%, *P*=.047). Sixty‐three per cent (501/795) of EV cases had co‐infections with other respiratory viruses and bacteria. RSV and AV (21.5% and 30.6%, respectively) were the most frequently detected viral co‐infections with enterovirus. The majority of patients (70.0%, 582/831) had a recorded fever, and 25% (211/829) of cases had symptoms of ≥3 days prior to admission. Almost all enterovirus‐associated SARI cases (93.4%, 781/836) had antibiotics prescribed on admission, and the majority (74.5%, 618/829) were hospitalized for less than 3 days. The in‐hospital case fatality rate was 2.2% (19/840). However, these deaths could not be attributed solely to enterovirus infection as these patients were also co‐infected with various other pathogens: 15.8% (3/19) *Mycobacterium tuberculosis*; 11.8% (2/17) adenovirus; 10.5% (2/19) human metapneumovirus; 15.8% (3/19) parainfluenza virus 2; 10.5% (2/19) parainfluenza virus 3; 15.8% (3/19) respiratory syncytial virus; and 5.3% (1/19) *Pseudomonas aeruginosa*,* Staphylococcus aureus* and *Streptococcus pneumoniae* (in blood samples).

**Table 1:** Clinical and epidemiological characteristics of patients hospitalized with enterovirus‐associated severe acute respiratory illness at surveillance sites, South Africa, 2009‐2011

Variables	All SARI samples n/N (%)	All enterovirus‐positive samples n/N (%)	Enterovirus serotyped n/N (%)	Species A n/N (%)	Species B n/N (%)	Species C n/N (%)	Species D n/N (%)	*P* value
Demographic and clinical characteristics								
Age (in years)								
<1	4263/13 598 (31.4)	377/839 (44.9)	23/45 (51.1)	3/7 (42.9)	10/20 (50)	5/8 (62.5)	5/10 (50)	.836
1‐4	2411/13 598 (17.7)	349/839 (41.6)	19/45 (42.2)	4/7 (57.1)	8/20 (40)	2/8 (25)	5/10 (50)	
≥5	6924/13 598 (50.9)	113/839 (13.5)	3/45 (6.7)	0/7 (0)	2/20 (10)	1/8 (12.5)	0/10 (0)	
Male Sex	6640/13 602 (48.8)	489/839 (58.2)	21/45 (46.7)	4/7 (57.1)	9/20 (42.9)	3/8 (37.5)	5/10 (50)	
Year								
2009	3679/13 656 (26.9)	315/3128 (10.1)	8/45 (17.8)	1/7 (14.3)	4/20 (20)	2/8 (25)	1/10 (10)	.047
2010	4613/13 656 (33.8)	200/3460 (5.8)	24/45 (53.3)	6/7 (85.7)	11/20 (55)	5/8 (62.5)	2/10 (20)	
2011	5364/13 656 (39.3)	327/3672 (8.9)	13/45 (28.9)	0/7 (0)	5/20 (25)	1/8 (12.5)	7/10 (70)	
Symptoms ≥3 d prior to admission	6867/13 421 (51.2)	211/829 (25.5)	9/45 (20)	1/7 (15.3)	7/20 (35)	1/8 (12.5)	0/10 (0)	.125
Fever (≥38°C)	10 755/13 547 (78.4)	582/831 (70)	31/45 (68.9)	3/7 (42.9)	16/20 (80)	4/8 (50)	8/10 (80)	.158
Oxygen therapy	4491/13 508 (33.3)	236/831 (28.4)	15/45 (33.3)	2/7 (28.6)	6/20 (30)	1/7 (12.5)	6/10 (60)	.215
Antibiotics prescribed on admission	12 903/13 549 (95.2)	781/836 (93.4)	41/45 (91.1)	7/7 (100)	17/20 (85)	8/8 (100)	9/10 (90)	.809
Hospitalization (≥3 d)	9053/13 482 (67.2)	362/832 (43.5)	18/45 (40.9)	1/7 (14.3)	11/20 (55)	2/8 (25)	4/10 (44.4)	.236
In‐hospital death	755/13 526 (5.6)	19/840 (2.2)	2/45 (4.4)	0/7 (0)	1/20 (5)	1/8 (12.5)	0/10 (0)	.606
Underlying medical conditions								
HIV	5264/10 666 (49.4)	107/541 (19.8)	4/34 (11.8)	0/5 (0)	4/17 (23.5)	0/3 (0)	0/9 (0)	.412
Pre‐maturity^a^	178/6661 (2.6)	21/725 (2.9)	2/45 (4)	0/7 (0)	1/20 (5)	1/8 (12.5)	0/10 (0)	.606
Other medical conditions^b^	1189/13 588 (8.8)	62/838 (7.4)	4/45 (8.9)	0/7 (0)	3/20 (15)	1/8 (12.5)	0/10 (0)	.586
Co‐infections								
*Streptococcus pneumoniae* (blood)	706/10 242 (6.9)	23/505 (4.6)	2/31 (6.5)	0/7 (0)	2/14 (14.3)	0/4 (0)	0/9 (0)	.729
Respiratory syncytial virus	1897/13 413 (14.1)	181/842 (21.5)	5/45 (11.1)	0/7 (0)	2/20 (10)	1/8 (12.5)	2/10 (20)	.773
Para‐influenza 2 virus	148/13 413 (1.1)	20/842 (2.4)	2/45 (4.4)	0/7 (0)	2/20 (10)	0/8 (0)	0/10 (0)	.798
Influenza virus (A and B)	1168/13 430 (8.7)	33/842 (3.9)	2/45 (4.4)	0/7 (0)	2/20 (10)	0/8 (0)	0/10 (0)	.798
Adenovirus	1129/12 228 (15.8)	236/772 (30.6)	9/39 (23.08)	1/6 (16.7)	6/16 (37.5)	0/7 (0)	2/10 (20)	.246
Any viral coinfection	7052/12 860 (54.8)	501/795 (63)	19/39 (48.7)	3/6 (50)	10/16 (62.5)	1/7 (14.3)	5/10 (50)	.205

Abbreviations: ICU, Intensive Care Unit; HIV, human immunodeficiency virus.

a Pre‐maturity was classified as birth before 37 wks of gestation as reported on the road‐to‐health card.

b Other evaluated underlying medical conditions included: asplenia or sickle cell anaemia; chronic illness, including chronic lung, renal, liver or cardiac disease and diabetes; other immunocompromising conditions (excluding HIV), including organ transplant, primary immunodeficiency, immunotherapy and malignancy; neurological disorders and burns.

HIV status was known for 64% (541/842) of cases, among whom the prevalence of HIV was 19.8% (107/541). Close to 62% (8/13) of the cases with fatal outcome were HIV positive.

### 3.2 Molecular epidemiology of enterovirus species A to D

**Figure 1:**
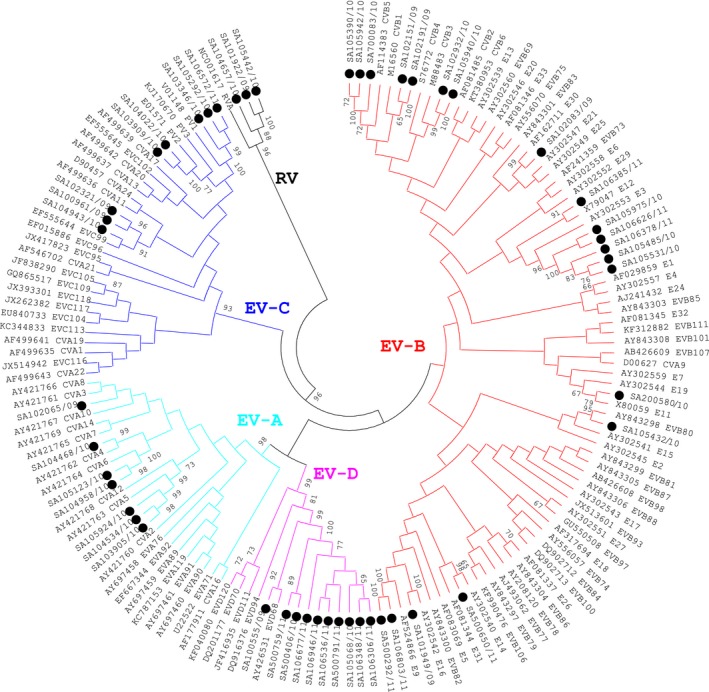
Molecular characterization of enterovirus clinical samples based on phylogenetic analysis of nucleotide sequences of the partial VP1 genomic region. Trees were constructed using neighbour‐joining methods as implemented in MEGA 5 software (http://www.megasoftware.net). Bootstrap values from 1000 replicates are shown on the nodes. Black dots=South African samples 2009‐2011

**Figure 2:**
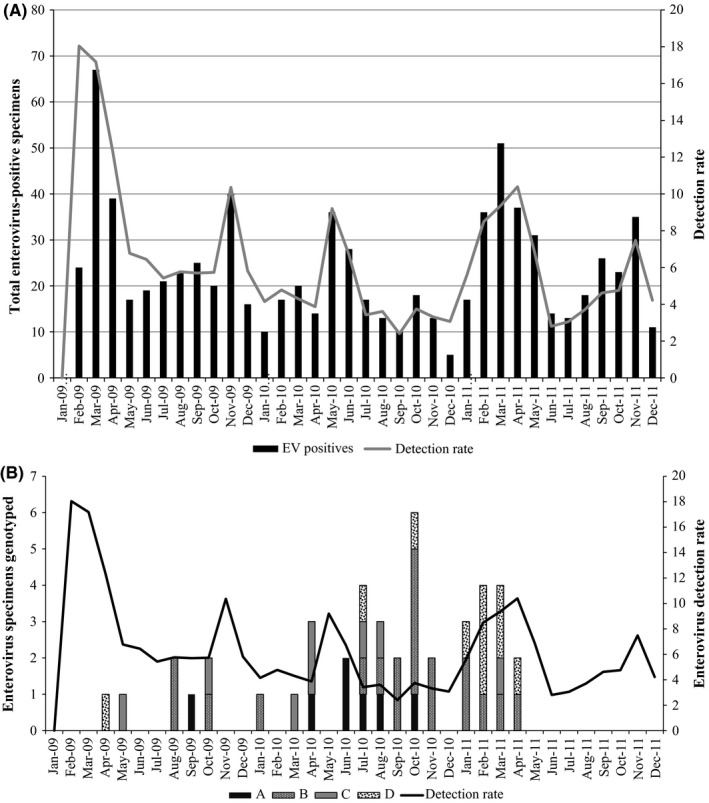
Epidemiologic graphs showing: (A) Distribution of enterovirus in South Africa during 2009‐2011 and (B) Distribution of enterovirus species detected in South Africa during 2009‐2011

In this study, 48 (24%) from the 200 randomly selected EV‐positive samples could be serotyped of which 3 grouped with the rhinoviruses; these were excluded from further analysis but are indicated as reference strains on the phylogenetic tree (Figure 1). EV‐B (44%, 20/45) and EV‐D (22%, 10/45) species were more prevalent than EV‐A (16%, 7/45) and EV‐C (18%, 8/45) with bootstrap support of 95‐99% for clusters (Figure 1). EVs were detected throughout 2009‐2011, with no evident seasonality, although a higher EV‐D68 activity was seen in 2011 (Figure 2A), whereas EV species B, C and D co‐circulated during 2009‐2011 (Figure 2B) and EV‐A was detected only in 2009 and 2010. The majority of EV‐A (86%, 6/7) and EV‐B (55.0%, 11/20) strains were detected in 2010, while EV‐C (62.5%, 5/8) and EV‐D (70.0%, 7/10) were more commonly detected in 2011 (Table 1). The majority of patients infected with EV‐B (80%, 16/20) and EV‐D (80%, 8/10) had a recorded fever. A high proportion of viral co‐infections (other than EV) were found among EV‐B (62.5%, 10/16)‐ and EV‐D (50%, 5/10)‐positive patients. All HIV‐infected patients were infected with EV‐B strains (Table 1, Figure 1).

### 3.3 Enterovirus serotypes and circulation of EV‐D68 clades during 2009‐2011

Seventeen distinct EV serotypes were identified of which EV‐D68 (22%, 10/45) and Echovirus 3 (E3) (11%, 5/45) were the most frequently detected. Other serotypes detected include coxsackie virus A,^3^, ^4^, ^5^, ^6^ coxsackie virus B,^1^, ^3^, ^5^ echovirus (9, 11, 12, 14, 16, 30, 80, 99) and poliovirus 1, 2 (Figure 1). Polioviruses were Sabin strains detected in the respiratory specimens of children that had recently been vaccinated.

**Figure 3:**
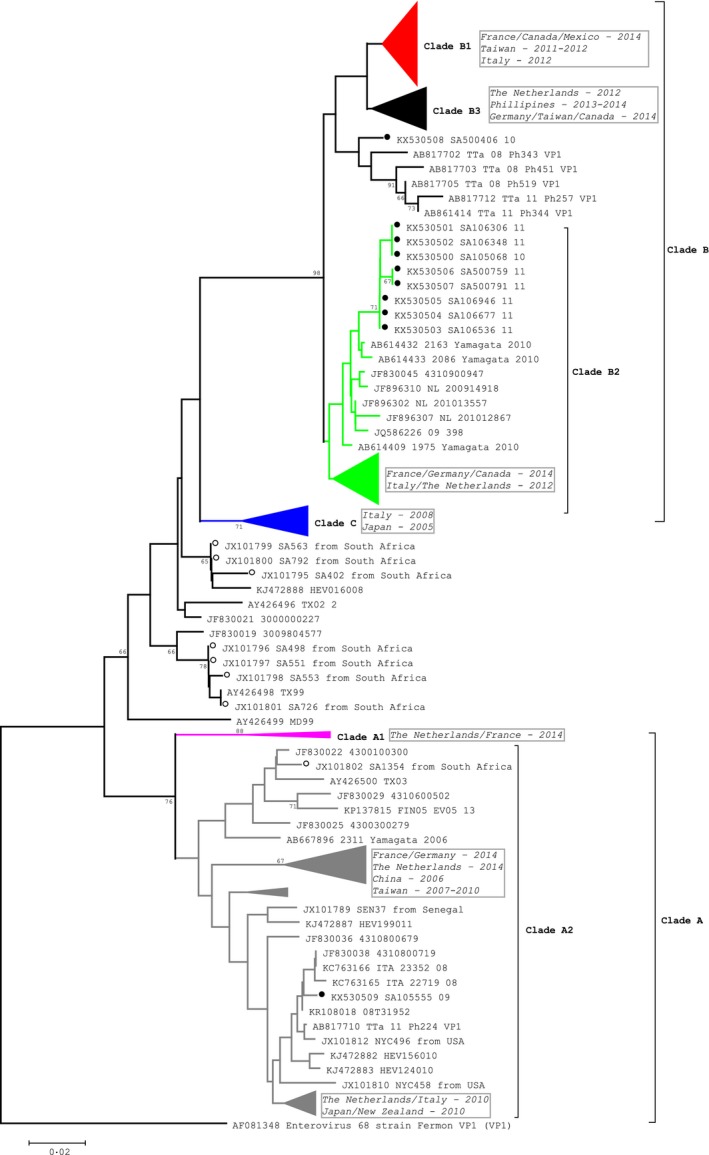
Molecular characterization of EV‐D68 clinical samples based on phylogenetic analysis of nucleotide sequences of the partial VP1 genomic region. Trees were constructed using neighbour‐joining methods as implicated in mega 5 software (http://www.megasoftware.net). Bootstrap values from 1000 replicates are shown on the nodes. Scale bar indicates number of nucleotide substitutions per site. Black dots=South African strains 2009‐2011; Open dots=South African strains 2000‐2001. Clades B1, B3, C A1 and partial B2 and A2 have been compressed to show comparison of South African strains to strains from other countries (country and year in blocks). Total number of sequences=312 (representative strains from 2000 to 2016)

Here EV‐D68 strains are grouped according to phylogenetic clades A, B and C. The majority of EV‐D68 strains (90%, 9/10) are in clade B. Previously reported South African EV‐D68 strains from 2000 to 2001 7 clusters in clades C and A (Figure 3). The South African clade B strains clustered with strains from respiratory specimens from Japan, 2010 and the Netherlands, 2009‐2010 (8/10) and from the Philippines, 2008 and 2011 (1/10). The South African clade A strain clustered with strains from respiratory specimens from Italy, 2008; the Netherlands, 2008; the Philippines, 2011; and Kenya, 2010. The designated clades B1 and B3 described recently were based on more complete gene sequence data (~930 base pairs for complete VP),^9^, ^10^ and therefor, our phylogenetic tree (based on 300 base pair VP1 fragment) did not have good bootstrap support for clade B1 and B3.

No neurological disease or paralysis symptoms were reported or available for patients from this study who were positive for EV‐D68. Demographic and clinical characteristics of patients in whom EV‐D68 were isolated are described in Table 2. Ages ranged from 3 to 54 months and respiratory symptoms included cough, chest indrawing, breathing difficulty and tachycardia. No amino acid changes in the VP1 region that could lead to increased virulence 21 were observed in the South African samples (results not shown).

**Table 2:** Demographic and clinical characteristics of ten patients in whom EV‐D68 was identified in South Africa

Characteristics	SA500759/11	SA106306/11	SA105068/10	SA106946/11	SA106677/11	SA106348/11	SA500406/10	SA500791/11	SA106536/11	SA100555/09
Demographic characteristics										
Age (in months)	25	15	3	11	3	16	54	16	11	5
Sex	F	F	F	M	M	F	M	M	F	M
Year	2011	2011	2010	2011	2011	2011	2010	2011	2011	2009
Medical history										
Underlying illness	N	N	N	N	N	N	N	N	N	N
HIV infected	N	N	N	N	N	N	N	N	UNK	N
Clinical presentation and course										
Symptoms (days) prior to admission	1	1	1	1	0	1	2	1	1	0
Fever (≥38°C)	Y	Y	Y	Y	N	Y	Y	Y	Y	UNK
Respiratory symptoms	C, B, S, L, A	C, B, CI, T	C, B, T, A, V	C, T	C, CI	C, B, T	C, B, CI, S	C, B, T	C, B, CI, S, T, A, V	C, B, CI, T

Abbreviations: M, Male; F, Female; N, No; UNK, Unknown; Y, Yes; C, Cough; S, Stridor; R, Rhinorrhoea; M, Malaise; F, Fatigue; A, Anorexia; L, Lethargy; B, Breathing difficulty; CI, Chest indrawn; T, Tachycardia; V, Vomiting.

## 4 Discussion

We described the EV species circulating among patients from all age groups with SARI in South Africa. During 2009‐2011, EV was detected in 8.2% of hospitalized SARI patients, which is within the range reported in other studies (3‐25%).^22^, ^23^, ^24^ Seventeen EV serotypes were identified representing all four EV species. EV‐D68 was detected in 22% of EV cases characterized, which compares to frequencies reported in studies from the Netherlands (25%) and Germany (7.7%).^25^, ^26^ The EV‐D68 strains that circulated in South Africa in 2009‐2011 clustered in clades A and B of the EV‐D species with the majority of strains grouping in clade B. All EV‐D‐positive patients in this study were less than 5 years of age, which is similar to the typical younger age distribution reported for EV.^22^ A study to determine the worldwide emergence of EV‐D68 included eight South African EV‐D68 strains identified in hospitalized children over a one‐year period (2000‐2001).^7^ One of these South African strains (2001) was found within clade A, while the remaining seven (2000‐2001) formed a cluster with sequences from Europe and the USA ancestral to clades B and C.^7^ More recent samples (2009‐2011) were predominantly located in clade B of EV‐D68.

It is speculated that the 2014 EV‐D68 outbreak was likely due to modifications of the receptor interaction affecting the local cell tropism; the inability of previous established anti‐EVD68 antibodies to detect the altered amino acid residues of the VP1 gene.^21^

Recent experience indicates that EV‐D68 may be associated with a more severe clinical presentation than other EV strains.^27^ However, although the South African patients with EV‐D68 detected in this study were all hospitalized, none were admitted to ICU or died. We did not have any recorded symptoms of neurological disease or paralysis for these patients, and no causative link between EV‐D68 infection and neurological disease has been established to date.^25^ No EV‐D‐positive samples were obtained from a South Africa study characterizing non‐polio enteroviruses in stool and cerebrospinal fluid samples of patients symptomatic for gastroenteritis and acute flaccid paralysis, respectively (personal communication, Wayne Howard, National Institute for Communicable Diseases). Higher EV‐D68 activity in 2011 is consistent with another report.^28^

Our study has limitations that merit discussion. Only 24% of EV‐positive samples selected for this study could be serotyped. The low success rate for genotyping may be due to low viral loads in clinical samples (Ct values >35 on original real‐time PCR assay) and subsequent inefficient nested amplification and sequencing of variable regions for phylogenetic analysis by degenerate PCR primers used for direct sequencing of PCR products. Furthermore, our study was limited to patients hospitalized with SARI and the presence of neurological manifestations was not specifically explored at time of participant enrolment. Identification of some EV positives as rhinoviruses by molecular characterization could likely be due to cross‐reactivity in the 5` UTR due to sequence homology between EVs and rhinoviruses. Further investigation is needed to determine the disease association of EVs and specifically EV‐D68 infection with non‐respiratory illness as well as full genome sequencing to determine possible recombination of EV‐D68. As this study did not include an asymptomatic control group, we were limited interpreting the attributing causality to enteroviruses in the presence of high‐frequency co‐infections as well as determining concurrent prevalence in the healthy population.

In conclusion, we showed enteroviruses frequently contributed to SARI in South Africa. A high diversity in the EV species that circulated in South Africa during 2009‐2011, EV‐D68 specifically circulated at high frequencies among children <5 years of age. EV should be closely monitored to assess the emergence of highly pathogenic strains. There is no vaccine or antiviral available for non‐polio EVs. Understanding the contribution of EV serotypes to severe illness will allow for informed decision‐making on potential candidate vaccines and development of therapeutic interventions.

## Conflict of Interest

Cheryl Cohen reports grants from US Centres for Disease Control and Prevention, during the conduct of the study. Halima Dawood has received honoraria from Pfizer‐South Africa, Novartis‐South Africa and MSD‐South Africa for speaking engagements; and travel grants from Novartis‐South Africa and Myalan‐South Africa. Shabir Madhi reports grants from Centre for Diseases Control, USA, during the conduct of the study; grants from Novartis; grants and personal fees from GSK; grants and personal fees from Pfizer; personal fees from Medimune; personal fees from BMGF, outside the submitted work.

## Disclaimer

The findings and conclusions in this report are those of the authors and do not necessarily represent the official position of the US Centers for Disease Control and Prevention.
